# Lower-risk substance use guidelines accessible by youth

**DOI:** 10.1186/s13011-023-00516-3

**Published:** 2023-02-13

**Authors:** Zakkaery R. Moebes, Kiffer G. Card, Brett Koenig, Cecilia Benoit

**Affiliations:** 1 Canadian Institute for Substance Use Research, University of Victoria, 2300 McKenzie Ave, BC Victoria, Canada; 2 Faculty of Health Sciences, Simon Fraser University, Burnaby, BC Canada

**Keywords:** Substance use, Youth, Guidelines, Digital assessment, Harm reduction

## Abstract

**Background:**

Lower-risk substance use guidelines (LRSUGs) are an evidence-based harm reduction strategy used to provide information to people who use drugs so they can reduce harms associated with substance use.

**Objectives:**

This study aimed to identify LRSUGs accessible to youth and to characterize the recommendations within these guidelines. The overall goal is to identify gaps in current LRSUGs and to inform researchers and policymakers of the kinds of health information youth can access.

**Methods:**

We conducted a digital assessment using the Google search engine to identify LRSUGs that could be identified by youth when searching for official sources of information related to commonly used substances, including cannabis, caffeine, alcohol, hallucinogens, prescription opioids, nicotine, and/or prescription stimulants. LRSUGs were coded and data were extracted from them to identify gaps.

**Results:**

One hundred thirty LRSUGs were identified; most focused on alcohol (*n* = 40, 31%), cannabis (*n* = 30, 23%), and caffeine (*n* = 21, 16%). LRSUGs provided recommendations about dosing (*n* = 108, 83%), frequency of use (*n* = 72, 55%), and when to use (*n* = 86, 66%). Most LRSUGs were published by health (*n* = 51, 39%) and third-sector organizations (n = 41, 32%), followed by provincial/state (*n* = 18, 14%), government (*n* = 14, 11%), municipal (*n* = 4, 3%), and academic (*n* = 2, 2%) sources. Only 16% (*n* = 21) of LRSUGs were youth-specific and one-quarter (*n* = 32, 25%) of LRSUGs provided gender-specific recommendations. Most guidelines featured information on short (*n* = 76, 58%) and long-term (*n* = 69, 53%) negative effectives and positive effects of substances (*n* = 56, 43%). Less than half (*n* = 50, 38%) of LRSUGs cited evidence in support of the information they provided.

**Conclusions:**

We identified several areas in the current LRSUGs for youth that need to be addressed. Among the gaps are a lack of LRSUGs developed specifically for youth, a lack of youth engagement in developing harm reduction strategies centered around them, and a lack of evidence-based LRSUGs. Youth-oriented, evidence-based LRSUGs are needed to better support youth who use substances and help them manage the negative effects of substance use.

**Supplementary Information:**

The online version contains supplementary material available at 10.1186/s13011-023-00516-3.

## Introduction

Substance use is common among Canadian youth, defined by the United Nations as individuals 15 – 24 years of age [1]. According to Health Canada [2], approximately 20% of youth use cannabis and e-cigarettes and twice as many drink alcohol. Young [3] found that between 4.2–7.7% of Canadian youth use psilocybin and between 3.7–4.3% of youth use LSD. Youth use these and other substances to achieve desirable effects, such as eliciting euphoric experiences, facilitating social inclusion, and coping with life events [4, 5]. Substance use can also lead to negative health outcomes, including cardiovascular and/or cognitive distress, addiction, and respiratory disease [6, 7]. Government organizations have focused on the negative effects of substance use among youth, and most continue to recommend that youth not use drugs. Focusing purely on abstinence ignores the fact that many young people continue to use substances. While these abstinence recommendations are supported by existing evidence, the continued widespread consumption of substances highlights the need for further harm reduction strategies to support young users.

Lower Risk Substance Use Guidelines (LRSUGs) are tools that provide information regarding evidence-based harm reduction strategies that can help people who use drugs navigate the risks associated with substance use [6, 8–10]. LRSUGs provide recommendations that empower youth to make better choices about their substance use by providing them with targeted strategies within their control that allow them to tailor their substance use patterns in a healthier fashion [6, 8–10]. Lee et al. [10] demonstrated that most Canadian adults follow the recommendations provided by the government but found that the notable exception was the abstinence-oriented guideline regarding smoking cannabis. In Canada, official LRSUGs have been developed and evaluated for adult alcohol and cannabis use, with other jurisdictions developing their own guidelines accordingly [5, 9, 11]. LRSUGs are published in government sources, researchers’ peer-reviewed articles, and documents produced by health organizations and third sector groups. Unlike government sources, health organizations (i.e., organizations who primarily focus on public health and well-being) and third sector groups (i.e., groups that do not fit into the other categories) will frequently publish LRSUGs and harm reduction material for substances which are criminalized for certain populations, including youth under age 18, and drugs that may be used for a different purpose than directed (reference?).

There is some evidence that LRSUGs should be tailored for key populations. Batty et al. [12] report that men were more likely than women to exceed both the daily and weekly recommended alcohol guidelines – suggesting that the development and promotion of LRSUGs should consider gender-based differences [13, 14]. There is also a paucity of research on age specific populations. To our knowledge, existing studies on LRSUGs have focused almost exclusively on those designed for the adult general population. While there are a few notable exceptions, the dominant recommendation for youth is abstinence. For example, abstinence is the only recommendation for youth in Canada’s official Lower Risk Cannabis Use Guidelines [5]. While cannabis and other drugs can put youth at risk for serious health harms [7, 15], LRSUGs are nevertheless needed to ensure that when youth choose to use substances, they have the accurate information they need to minimize potential harms.

Existing evidence related to harm reduction among youth suggests that harm reduction strategies are effective and can create opportunities to engage youth in treatment and care [16]. Substance use can directly affect a youth’s cognitive development including their memory, attention, and learning abilities [15]. Youth-specific harm reduction strategies, such as LRSUGs, are critical for young people. This is especially important when considering how information should be tailored and targeted so that youth find it helpful.

Given the potential for LRSUGs to help youth mitigate the negative effects of substance use, we conducted a digital assessment to identify and characterize LRSUGs for widely used substances and/or easily accessible substances consumed recreationally (i.e., cannabis, alcohol, caffeine, hallucinogens, nicotine, prescription opioids, and prescription stimulants) that are accessible to Canadian youth. In doing so, we sought to identify [1] which organizations were producing LRSUGs, [2] what drugs were LRSUGs developed for, [3] what information was being provided (e.g., information about substance legality, long- and short-term effects of the substance, dosing, timing, and frequency of use), and [4] whether guidelines were being tailored for key populations (e.g., youth, sexual and gender minorities). Our end goals were to help public healthcare practitioners and researchers better understand the health information available to youth online and highlight any information gaps that can be addressed in future studies.

## Methods

### Search strategy & definitions

**Fig. 1:**
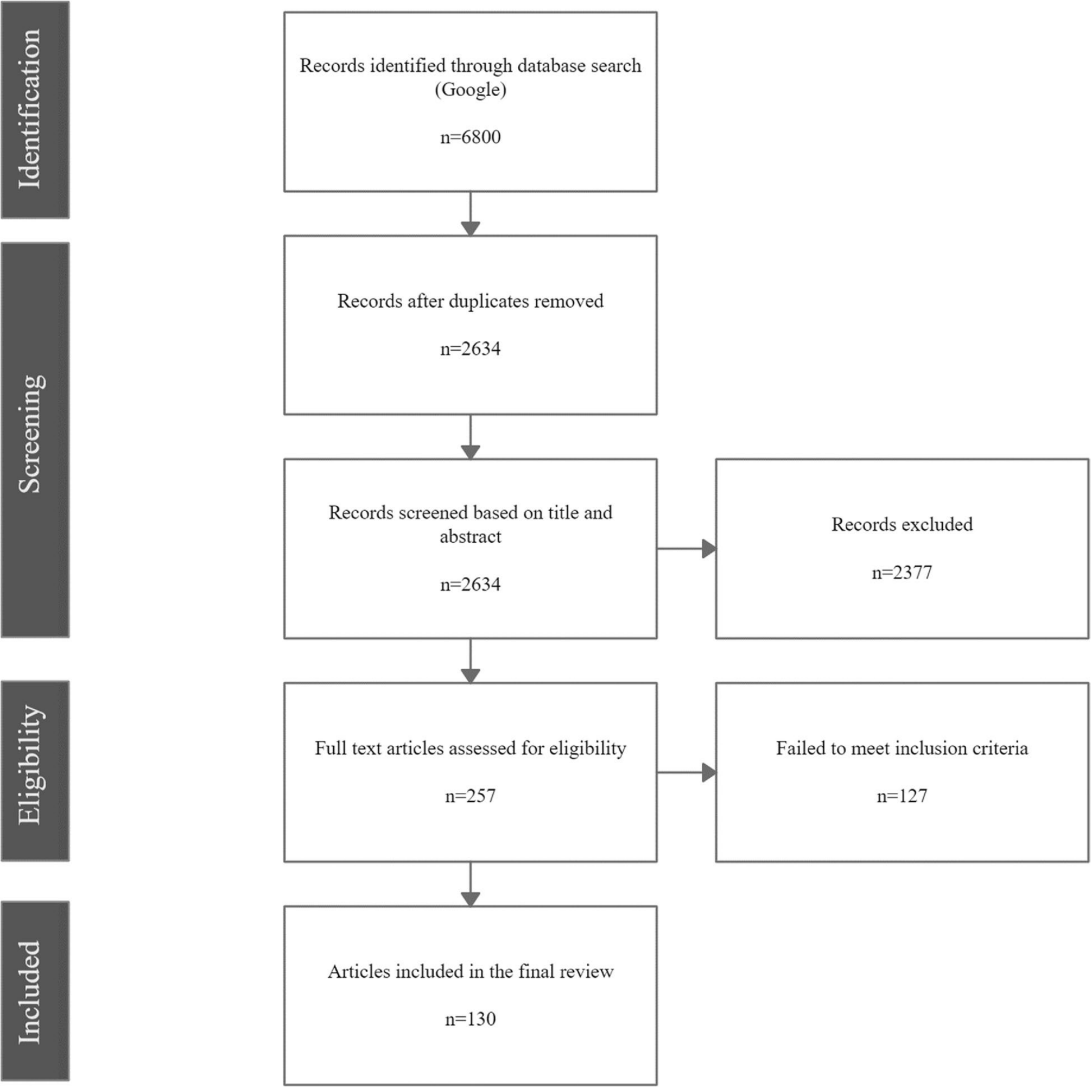
Lower-risk substance use guidelines accessible by youth prisma diagram

To identify LRSUGs accessible to youth, we conducted a digital environmental scan and assessment of existing LRSUGs’ available to youth. We defined LRSUGs as any set of recommendations designed to help substance users identify and modify behavior to manage the negative effects of using substances [6, 8–11]. Table 1 provides a list of the LRSUG definitions that we used for this study. To accomplish the digital environmental scan, we devised a novel method of searching the grey literature which was based on a *rapid review framework* [17, 18]. We streamlined our review methods by restricting our search to the first five pages of Google. This decision was made because we wanted a search process that would return results that youth might realistically encounter while looking for health information about substances. The use of the first five pages in Google was supported by previous research showing that the first few search results within search engines are a widely used source of health information among youth and has been used in previous studies [19]. This study was also designed to focus on information available through an internet search. We excluded other platforms (e.g., social media and physical distribution) primarily due to the difficulty in finding reliable predicable information on these platforms. Our review consisted of five steps (See Fig. 1).

**Table 1:** The List of Definitions for Lower-Risk Substance Use Guideline’s Used in this Study

Lower-Risk Substance Use Guideline Definitions
“The LRCUG are based on scientific evidence, identifying behaviors within the user’s control that influence the risk of health consequences from cannabis use. Our expert group systematically reviewed up-to-date evidence, and translated it into concrete recommendations on how to practically reduce such health risks.” [6]
“An important educational tool in a public health-oriented alcohol policy are so-called ‘Low Risk Drinking Guidelines’.^7^ These use scientific evidence to provide guidelines on practices or patterns of alcohol use that substantially reduce the risks of experiencing acute and long-term harms.^7^” [8]
“… evidence consistently shows that individual substance use behaviors, and corresponding choice-making by users, substantially influence related health – and, on the population level, public health – outcomes. Hence, informing and influencing individual users to make choices to lower substance use-related health risks, based on scientific evidence, constitutes an integral component for a public health approach.^44^ “ [9]
“… the LRCUG present a set of user-oriented recommendations towards informing and adjusting use-related risk behaviors, and consequentially reducing acute or long-term health harm for desired results. As such, the LRCUG serve as a ‘targeted prevention’ tool, as exists in other areas of health behaviors (e.g., low-risk drinking, safer sex, healthy eating/ nutrition guidelines) (Johnson et al., 2003; Mozaffarian, 2016; Rehm and Patra, 2012; Snook, 2004).” [10]
“It was assumed at the outset of this undertaking that guidelines which set specific low-risk levels are a useful device to assist consumers in making individual drinking decisions.” [11]

### Google search & article extraction

In Step 1, we conducted a series of Google searches, using the search terms and phrases listed in Supplemental Table 1. Search terms were generated by using a smaller scale preliminary search, with search terms that lead to the largest number of relevant LRSUGs being selected for inclusion in the study. Given that Google’s search algorithm incorporates geographic location and search histories, this search was conducted using Incognito mode and with cookies turned off to limit any potential impact our specific geographical location on the search results [20]. We included LRSUGs from other jurisdictions due to using Google as our search engine. In Step 2, we extracted the returned search results from the first five pages of Google search, resulting in fifty links for each search term.

### Inclusion & exclusion criteria

In Step 3, the titles of each search result were reviewed, and documents not related to using the substance were excluded (e.g., cultivation guidelines). In Step 4, we visited the web pages for the returned results and applied our exclusion criteria. LRSUGs were excluded if they [a] were not written in English, [b] did not have any LRSUGs; [c] did not contain any information related to the low-risk substances we identified for this study; [d] were not accessible (e.g., due to a paywall); or [e] if they contained abstinence only recommendations. We did not exclude LRSUGs on the sole basis of not containing youth specific information because youth can still access and use the information in these LRSUGs.

### Guideline coding

In Step 5, we coded and extracted information from the identified LRSUGs. Variable codes were identified a priori. All codes were designed as categorical or binary. The codes were generated from the information contained within the LRSUGs identified in a preliminary search. There were 15 codes in total capturing information about [1] the organization publishing the LRSUGs (i.e., Health [e.g., public health units], Government, Provincial, Municipal, Academic, Third-Sector [e.g., a retailer or drug user union]), [2] the drugs included in the LRSUGs, [3] whether the guideline was tailored, or partially tailored, for youth, [4] whether the guideline cited evidence in support of its recommendations, [5, 6] whether the information contained tailored information for key populations and by gender, [7–9] whether the LRSUGs provided recommendations about dosing, frequency of use, or timing of use, [10–13] whether the LRSUGs discussed the legality of substances, positive effects of use, negative short term effects and negative long term effects, [14] whether additional resources (e.g., link/phone number to a treatment service) were provided or recommended, and [15] whether the LRSUGs recommended abstinence. Extracted data was stored in an excel sheet, with each LRSUG representing one row and each code representing one column. Using these data, the number and proportion of LRSUGs that included each code were numerically calculated.

## Results

### Synthesis

A total of 136 Google searches were conducted, returning 6800 results. After removing duplicates, 2,634 unique documents were identified. Of these, 257 were identified as LRSUGs. Among the 257 identified LRSUGs, 127 were excluded because they did not meet our criteria, resulting in a final inclusion of 130 LRSUGs (See Lower-Risk Substance Use Guidelines for Youth—Guideline Coding Table—Additional File 1). Table 2 provides a summary of results. Most LRSUGs were published by health (*n* = 51, 39%) and third-sector organizations (*n* = 41, 32%), followed by provincial/state (*n* = 18, 14%), government (*n* = 14, 11%), municipal (*n* = 4, 3%), and academic (*n* = 2, 2%) sources. The most common LRSUGs related to alcohol (*n* = 40, 31%), cannabis (*n* = 30, 23%), and caffeine (*n* = 21, 16%). Only 2 (2%) of LRSUGs focused on prescription stimulants and only 5 (4%) focused on nicotine. Less than a fifth (*n* = 21, 16%) of the LRSUGs were youth-specific; while almost half (*n* = 58, 45%) were only partially tailored to youth (i.e., contains information specifically for youth, despite the public being the target demographic). Over half (*n* = 84, 65%) of the LRSUGs included recommendations for key sub-populations (i.e., pregnant women, people with a predisposition to mental health complication, people on medication, and people with physical conditions that may be made worse with substance use), but only 25% (*n* = 32) contained sex and gender specific recommendations. Recommendations included in the LRSUGs considered dosages (83% *n* = 108,), frequency of use (*n* = 72, 55%), and when to consume (*n* = 86, 66%). Less than one-fifth (*n* = 23, 13%) of LRSUGs recommended abstinence as a first line response. Just one-quarter (*n* = 32, 25%) of the LRSUGs provided information about the legality of substances, 43% (*n* = 56) discussed positive effects of using the drug, 58% (*n* = 76) discussed short-term negative effects, and 53% (*n* = 69) discussed long-term negative effects. Finally, 38% (*n* = 50) of the LRSUGs were supported with appropriately cited evidence and 39% (*n* = 51) provided links to external resources to support youth who use drugs. Example recommendations for cannabis use are displayed in Table 3, demonstrating significant variability and diversity across recommendations (e.g., dosing, timing of use, age of initiation).

**Table 2:** LRSUG Digital Assessment Results

Information in Guidelines Sampled	*N*	%
*Organizations Guidelines Originated From*		
Health	51	39
Government	14	11
Provincial	18	14
Municipal	4	3
Academic	2	2
Third Party	41	32
*Drugs Covered*		
Alcohol	40	31
Caffeine	21	16
Cannabis	30	23
LSD	8	6
Nicotine	5	4
Prescription Opioid	8	6
Prescription Stimulant	2	2
Psilocybin Mushrooms	16	12
*Youth Specific Guidelines*		
Yes	21	16
Partially	58	45
*Recommendations*		
Cited Sources	50	38
Contained Legality Information	32	25
Contains Sex/Gender Information	32	25
Sub-Population Information	84	65
Dosing Information	108	83
Abstinence Recommendation	17	23
Frequency of Use Information	72	55
When to Use Information	86	66
Contains Additional Resources	51	39
Positive-Effect Information	56	43
Short-term Negative Effect Information	76	58
Long-term Negative Effect Information	69	53

**Table 3:** Example Guidelines for Youth’s Cannabis Use

Category	Sub-Category	Focus	Example Guideline Components
Abstinence	General	Abstinence	The best way to avoid the negative effects of cannabis is to abstain from its use
	Specific	Age of Onset	Younger users are more likely to experience harm because their brain is developing. It is best to delay use past age… (16 + , 18 + , 21 + , 25 + , late 20 s)
		Mental	Keep a check on your mental health and motivation and avoid using cannabis as your main way of having fun or coping with stress. There are healthier ways to enjoy yourself or deal with negative moods. If you think marijuana is affecting your mental health or motivation, ease off using it
		Mental	If you have a distressing mental experience while using cannabis, stop consuming it temporarily and seek help
		Remedies	If you are feeling too high some ways to reduce the high are to consume black peppercorn, CBD, stay hydrated and nourished, ibuprofen, smelling limonene terpenes, and to breathe deeply
		Situational	Know how cannabis affects you and know your limits. If it makes you tired or distracted, do not use it if you need to be alert and focused. Do not use before work or school unless you have a valid medical reason to do so. Employers have the right to expect their employees not to be high, stoned, or drunk on the job
		Situational	Frequent users who experience difficulty controlling their use should attempt to cease use; if they are unable to do so unaided, they should seek professional help
		Sub-Population	There are some populations at probable higher risk for cannabis-related adverse effects who should refrain from using cannabis, including: Users with a personal or family history of mental health problems; pregnant women; users with a personal or family history of mental health problems and pregnant women; users with a personal or family history of mental health problems, middle-aged men with cardiovascular issues, and pregnant women
		Synthetic Cannabis	Synthetic cannabis can lead to more acute and severe adverse health effects (including instances of death) compared to non-synthetic cannabis. The use of these products should be avoided
Behaviour	Legality	Combination	Do not break more than one law at a time. For example, if you are driving with cannabis in your car, make sure your lights are working on your vehicle and your registration is up to date
		Environment	Do not smoke in a car. The smell of cannabis emanating from a car is the single most common way people get busted. In fact, it is safest to keep it in the trunk, out of sight in an odor-proof container, such as a glass jar or an oven roasting bag. Don't use car ashtrays to hold your roaches or pipes
		Growing	Do not grow it unless you are legally allowed too. Stay within legal limits and keep the plant count as low as possible
		Legal Education	Educate yourself about your rights, health risks, laws, and consequences of using
		Mercantilism	Do not sell cannabis; every customer is a potential narc or snitch. Do not smuggle (including shipping cannabis through the mail illegally). Getting caught crossing international borders creates more serious problems than it is worth. The penalties for sales and smuggling are very serious
		Possession	Know the Legality, and make sure you are within your possession limit
		Sharing	Do not share with minors, cannabis is for adults
	Preparation	Food and Drink	Cannabis can make you hungry, so load up on your favorite snacks beforehand. Bring water, cannabis can prevent your body from producing saliva, leading to dry mouth; it can help with thirst, headaches, fatigue, and coughing. Always have 16 oz of water ready before you begin
		Hygiene	Keep your stuff clean. Keep your bongs and pipes clean and do not roll your weed on dirty surfaces. If sharing, hold joints or devices in a way that you can inhale the smoke or vapor without touching them to your lips. If sharing, quickly apply flame to the pipe mouthpiece or wipe with rubbing alcohol to kill germs
		Information	Consult with dispensary employees on different options and products for your needs and always read the product label carefully before use
		Planning	Have a safety plan or contact in case you feel you are in trouble. If you are planning to use any substances, tell your friends what you’re taking and how much. If anything goes wrong, they are equipped with the necessary information to tell medical personal
		Pre-Use Preparation	Eat a good meal at least three hours before a party. A full stomach can moderate some of the negative effects of cannabis. This reduces your chances of nausea and serves as protection for the stomach if you plan to use substances
		Responsibilities	Clear your schedule, weed smokers often pass out or feel heavy-bodied. You will in no way be able to function for regular routines after smoking weed. Do not blame cannabis for not achieving your goals, or for a lack of self-control. If you are, assess how you are using it. If it gets in the way of fulfilling obligations and responsibilities to yourself and your loved ones, consider re-assessing your usage
		Sexual Wellness	Carry condoms and lube
	Safety	Carpooling	Do not ride in a vehicle with someone driving who has recently used cannabis
		Combination	Do not break more than one law at a time. For example, if you are driving with cannabis in your car, make sure your lights are working on your vehicle and your registration is up to date
		Combination	Combining risky behaviour will magnify the risk of negative outcomes from using cannabis
		Driving	Do not drive or operate machinery while under the influence. Make sure you have money for a cab, bus rides or designate a sober driver. Wait (15 min, 1–2 h, 3 h, 5–8 h, etc.)
		Immune Wellness	If consuming with others, try not to share the smoking device. Sharing items that have touched your lips increases the risk of spreading infections including meningitis, flu, and other germs
		Injury Prevention	Prevent burns on your lips or fingers. Use a small piece of rolled unbleached cardboard as a filter. Avoid using cigarette filters—they do not remove toxins in the smoke
		Overdose Response	If you feel too high, don' t panic, acknowledge it as anxiety, eat, hydrate, and find a safe place, and distract yourself (a friend can help with this, and maybe talk you down), remember that nothing bad is going to happen. Effects wear off within 2–8 h
		Legality	If you are 19 years or older, possessing up to 30 g of cannabis for your own use is legal in Canada. Cannabis is regulated by the province of BC. You must be 19 or over to purchase, possess or use cannabis or cannabis products. Minors in BC (people under the age of 19) are not allowed to possess any cannabis. Be sure you know where and when it is safe and legal to use cannabis​
		Serious Medical Issue	Seek medical attention if the person is unconscious and cannot be wakened, their breathing is irregular and/or shallow, their skin is clammy or pale, or there is blood in their vomit. Place the person on his/her side, with one arm extended above the head (recovery position)
		Subscription Interaction	If you are using prescription medications, herbal supplements, or other products, or have health concerns, you should speak to your doctor or another medical professional before using cannabis. Avoid using if on any medication, herbal supplements, or other products that interacts with Cannabis
	Set and Setting	Set	Be clear about why you want to use. Is it going to help you in some way or make things worse?
		Setting	Only in smart and safe contexts. Trying cannabis at a weekend party is less likely to result in trouble or harm than smoking cannabis on school property or driving after using. Making informed decisions about where and with whom we use cannabis helps to minimize harms. It is also a good idea to have a responsible adult present who is not under the influence of liquor or drugs
	Social	Communication	Listen to the advice and criticism of others. If you are exhibiting behaviour that makes them uncomfortable, cannabis use may need to be reassessed
		Disposal	Do not dispose of used joints on the ground where animals or kids might find them
		Environment	Respect others: Do not smoke in designated non-smoking areas. Limit exposure of secondhand smoke to others. Do not smoke around children or if you are responsible for watching children
		Support	If you need more information or support, talk to your parent/guardian, teacher, coach, or other trusted adult
	Sourcing	Edible Quality	Shop at a legitimate dispensary that sells lab-tested edibles with labels that say how much THC is inside. Some edible products may have expiry dates and ingredient that can cause allergic reactions. The label should always be checked before consuming
		Extract Quality	For Extracts looks for products with less than 10% THC (100 mg/g) and higher or equal levels of CBD
		Product Quality	Care about quality. Whenever possible, choose organic cannabis products. Make sure the cannabis is from a reliable source, unadulterated, pesticide free and carefully assess the quality
		Strain Selection	Change the cannabis variety if the one you are using seems to be losing its effectiveness. Take note of what effect each variety produce for you (therapeutic and side effects); keeping a log can be helpful
	Storage	Storage	To avoid accidental overdoses with children or pets, store edibles and other cannabis products safely and out of reach. Do not leave open containers in the car
Pattern of Use	Administration	Bong/Pipe Administration	Stick to glass, stainless steel or brass bongs and pipes. Avoid wood, aluminum, rubber, and plastic bongs and pipes. Some can give off toxic fumes. Plastic bongs can contain chemicals like BPA and phthalates, which have been linked to serious health effects, including cancer. If you do use one, change the water frequently to limit exposure to germs and viruses
		Choice of Administration	The method of consumption and strain can affect dosage strength, effects, and the risks to the user. *(e.g., Smoking is one of the easiest ways to dose cannabis, but it can lead to respiratory harm. Edibles have no respiratory risk but can be difficult to dose due to the long onset time. Pills containing hash or cannabis oil or ingest *via* tincture or sprays; like edibles effects may take a while to kick in, start with no more than 2 drops and wait an hour before increasing dosage. Topicals are one of the safest ways to consume cannabis but may not result in psychoactive effects.)*
		Concentrate Administration	Dabbing concentrates is one of the cleanest ways to smoke cannabis. This is because you are inhaling vapor instead of smoke
		Ignition Source	To avoid inhaling unnecessary chemicals, use hemp paper coated with beeswax to light your cannabis rather than matches or a lighter
		Inhalation Techniques	Users should avoid deep inhalation, breath-holding, or other harmful smoking practices. Take smaller, shallower inhalations rather than deep inhales
		Paper Product Administration	Choose joints over blunts. Blunts can contain leftover carcinogens as well as potentially harmful chemicals themselves. They are also bigger, leading to a bigger dose
		Paper Product vs. Bong/Pipe Administration	Water bongs are not as safe as joints. Bongs filter out more THC than tars since water tends to absorb THC, and you can inhale water vapor or water drops into your lungs. This requires you to puff harder, increasing the amount of tar that is inhaled
	Dosing	Concentrate Dosing	Concentrate products frequently contain higher THC than other cannabis products, thus the risks associated with high THC content also apply to dabbing, and in some cases even more so. Products may also be impure and contain harmful substances
		Edible Dosing	Take your time with edibles. To avoid accidental overdose "start low, go slow". It takes longer to feel the effects of edibles than with other consumption methods *(e.g., 30 min—2 h., Start with a small amount and wait until you feel the effects before taking more. The full effects may take as long as 4 h to take effect. Start with an initial dose of 2.5 mg. Start with an initial dose of 10 mg or less. Start with an initial dose of 5 mg. Users should wait until they feel the effects before consuming more or wait until the next day; and increasing the next dose by 5-10 mg. Users should wait until they feel the effects before consuming more edibles. Users should wait at least 2 h before consuming more edibles.)*
		Extract Potency	For Extracts looks for products with less than 10% THC (100 mg/g) and higher or equal levels of CBD
		General Dosing	Start Low and Go slow, limit the amount of substance used and only start with a small amount. This is especially the case if you are using an unfamiliar cannabis product *(e.g., Wait at least an hour to gauge effects before consuming more. Wait to feel the effects before you take more, it takes seconds to minutes to feel the effects of smoking/vaping and 30 min—2 h for edibles)*
		High Potency	Use higher potency cannabis so you use less cannabis. Concentrates can be useful, particularly if you need higher doses. You can also use less and avoid unnecessary smoke and toxins in your lungs while still getting the same high
		New User	If you’ve never used cannabis before or have low tolerance starts with a lower THC product. If using an unfamiliar strain, sample a small amount first and wait to see how you react
		Product Strength	Higher strength cannabis can worsen the negative effects of cannabis and can lead to a higher chance of overdosing. Use cannabis with a lower THC content. It is also advisable to use cannabis with a high CBD:THC ratio
		Smoking Dosing	Smoke as little as possible. Effects are felt in seconds to minutes, it can take up to 30 min to feel the full effects *(e.g., Try 1 to 3 inhalations and wait 10 to 15 min to find the right dosage, increase dosage as necessary. Start with 1 or 2 puffs of a vape or joint with 10% (100 mg/g) or less THC*
	Intensity	Frequency of Use	Frequent use (i.e., daily, or near-daily use) is associated with most severe problems and should be avoided. Occasional use (e.g., use only on 1 day/week, weekend use only, etc.) at most
	Mixing	Mixing with Drugs	One at a time. Complications are more likely if you mix drugs. This includes alcohol, tobacco, pain medication, and street drugs. Be aware of any synergistic affect’s cannabis and any other drugs your taking has

## Discussion

### Primary findings

We conducted a digital assessment to identify and characterize LRSUGs for widely used substances (i.e., cannabis, alcohol, caffeine, LSD, psilocybin mushrooms, nicotine, prescription opioids, and prescription stimulants). In doing so, a number of key observations were made about existing LRSUGs. First, we observed that a variety of organizations were involved in the creation and publishing of LRSUGs for youth, with health organizations and third-parties accounting for the largest number of LRSUGs. Little research has examined what organizations and sources youth who use substances rely on the most when seeking information about substance use, nor if the presentation and format of LRSUGs from the organizations and institutions that produce these LRSUGs are acceptable and useful to youth. This is especially important with the emergence of platforms such as Tik-Tok and Twitter that may be underleveraged in existing knowledge translation strategies. Moreover, young people who use substances and access social media platforms for their health information may be exposed to various recommendations that come from other users. This information may be incorrect and could also be harmful to users. Future research should examine how both the publisher/platform and format affect the quality of information available and how these factors affect both the accessibility and uptake of LRSUGs.

Second, we note that the lack of recommendations tailored for age (the focus of our analysis), gender, physical disability, and mental health complications, reflects the need for multi-stakeholder approaches in setting standards for the development and promotion of these harm reduction tools that account for these specific populations of youth. Indeed, untailored LRSUGs can cause harm. Moore et al. [21] examined how well teenage drinkers follow low risk drinking guidelines, which recommended teens abstain for as long as possible, but that they should follow the adult guidelines if they do decide to drink. The authors found that low-risk teenage drinkers suffered a number of negative effects associated with high-risk alcohol use, despite their adherence to the lower-risk alcohol guidelines [21]. Similarly, given that substance use can affect specific populations (e.g., people predisposed to a psychiatric condition) more intensely, LRSUGs must consider their target audiences carefully [6, 7]. A particularly relevant finding was that only a few LRSUGs provided sex and gender-specific recommendations. Many substances will naturally affect people differently according to their biological sex and, in some cases, gender identification [13, 14]. Clearly it is important that future LRSUGs are made with specific populations in mind as one-size-fits-all approaches fail to accommodate the diverse needs of particular groups of youth.

To minimize the potential harms experienced by young people who use drugs, it is important to develop evidence-based LRSUGs that are specifically tailored to youth. Jenkins et al. [22], conducted a study investigating youth perceptions, experiences, and harm reduction strategies as they relate to cannabis; one major finding was the difference in these domains based upon geographic, cultural, peer, and political contexts. This is unsurprising considering that certain cultures may have traditions relating to certain substances. Given these realities, future research should evaluate how accessible and appropriate general LRSUGs are for different populations and demographic groups. It is necessary that stakeholders are engaged in the development of public health resources relevant to them by recruiting them for a variety of roles in the project, such as recruiting youth to be co-investigators on projects aimed at youth [16, 23–25]. This will lead to better evidence-based LRSUGs which are tailored to the specific needs of youth and will avoid any harms from unsuitable or non-evidence-based LRSUGs.

Third, we found that most LRSUGs related to cannabis, alcohol, and caffeine. Relatively few were available for prescription medications, LSD, psilocybin, and nicotine. These drugs are used by a sizable population of Canadian youth; approximately 10–30% use e-cigarettes, up to 7.7% use psilocybin mushrooms, 7% use prescription medications, and up to 4.3% use LSD [3, 26]. These substances also pose considerable risk if misused, most notably tobacco and prescription opioids [27, 28]. Marshall et al. [23] found that there are limited harm reduction strategies targeted towards young people who use prescription opioids. Similarly, Faraone et al. [29] found that most of the research regarding non-medical prescription stimulant use primarily focuses on college aged users, with very little information for youth users. LRSUGs are also needed for less normative/more stigmatized and less commonly used substances, given that finding information about these drugs from unofficial lay sources might be difficult for some users, and that information may not be evidence-based.

Fourth, the LRSUGs we reviewed presented inconsistent information. Recommendations about abstinence, dosing, timing of use, and frequency of use were common, but not always considered. For example, Table 1 provides a characterization of the recommendations found within various cannabis guidelines and demonstrates significant variation in recommendations regarding dosage, timing, age of onset, and consumption methods. Similarly, there was a lack of consistency in reviewing the positive and negative effects of substance use – including information about the criminality and legal risks associated with using drugs. These differences may arise from the ideological and evidentiary underpinnings of the LRSUGs and organizations developing them. Indeed, evidence from reliable academic sources was cited in less than half of the included LRSUGs. This is problematic given that youth may find it difficult to evaluate or trust the accuracy of information found online [30]. Providing solid scientific evidence and youth support for recommendations is critical to maintaining the integrity of LRSUGs while addressing the needs of youth.

Finally, we documented a clear missed opportunity for facilitating treatment and care, with less than half of LRSUGs providing links to external sources that could support youth who use drugs. Youth can take advantage of these links to get referrals to additional support, information, and treatment. Despite the advantages of these resources, youth may not be able to fully utilize them due to issues related to legal consent, program times, cost, access, and the appropriateness of the program for the individual. Presently, there is little research into if and how people who use substances understand the self-referral sections of drug education literature (e.g., brochures, pamphlets, LRSUGs designed to provide information on harm reduction) in search of treatment. Despite this, other harm reduction interventions have been found to increase the willingness for users to change their habits and increase the number of users entering other treatment programs [31, 32].

### Limitations

This study is limited by the difficulty of conducting searches within the grey literature. To conduct our study, we used a novel method of searching this area of literature. Indeed, there is a lack of a databases for LRSUGs for both youth and the general population. As such, our approach was designed to replicate the way an English-speaking youth would access this information and therefore may not be inclusive of all LRSUGs available on substance use. Therefore, a formal scoping review of all the LRSUGs available to youth was not undertaken as our review was meant to characterize and analyze the information ecosystem youth are accessing for knowledge on safe drug use. Given that most youth who search for information do so at a surface level and spend little time evaluating the information for its relevancy and accuracy, instead preferring rapid information [31], our approach to review the first few pages of each Google search aligns with the search patterns that a youth might typically engage in while still being comprehensive enough to generalize the content of these LRSUGs [21]. The language and search terms we used were also non-exhaustive and were limited to possible search terms a youth would use. We rationalized this decision with the large number of duplicates we encountered during our search. If a more comprehensive search terms were used, it is likely the percentage of duplicates to unique articles would be skewed even further due to the nature of Google.

We did not investigate the health information eco-system that exists on social media platforms. We rationalized this decision due to the constantly changing nature of social media, the difficulty in searching for specific information on the various platforms, the retrievability issues of prior found information, the known unreliability of information on these platforms, and the fact that visibility on these platforms is determined through popularity. Similarly, we did not investigate the health information and LRSUGs provided by small scale platforms and formats such as schools, religious groups, and community centers. Future studies should investigate the health information eco-system that exists on social media and the smaller scale platforms (e.g., school LRSUGs), and the differences, disadvantages, and advantages between the various platforms.

Our study also focused on the documents that were relevant to youth who currently use substances; thus, we excluded any documents that did not provide any recommendations to make substance use safer, such as those focused on abstinence only messaging. While this may be a limitation for our study, we justified this decision because of the anticipated difficulty in identifying the numerous sources that discourage youth from substance abuse, as well as the lack of useful information within these documents that pertain to youth substance users.

## Conclusion

Our study was designed to be a first step in developing better youth substance use LRSUGs by characterizing the information available to youth and identifying the gaps in this information. The results of our study highlight several implications for practice, policy, and health promotion. Based on our findings, despite legal age or substance legality concerns, we recommend that future LRSUGs adopt a harm reduction approach to youth substance use. There is also a need to develop more youth specific LRSUGs. Not only do more harm reduction strategies need to be developed specifically for youth but, when appropriate, these strategies should look for opportunities to adapt successful adult harm reduction strategies and develop youth-specific versions based off them. This is especially important as we found that most youth-specific LRSUGs are adapted from adult LRSUGs and fail to account for the distinct effects that drugs can have on youth and their development. In creating new LRSUGs, youth, clinicians, and researchers should be engaged. With youth being directly engaged in projects that are relevant to them, they will be better able to ensure that their needs are being properly addressed. Future LRSUGs must also be developed for other substances, including those which are criminalized and substances that are not as frequently used. Such LRSUGs should cite relevant research evidence and literature to enhance their validity and provide knowledge users with the ability to verify the accuracy of their recommendations. Evidence-based recommendations are also needed to provide information on dosing, frequency of use, timing of use, care and recovery, the user’s sex and gender, other demographic characteristics, and health status, if applicable. Finally, we stress that youth LRSUGs are not a one-size-fit-all approach, and that youth are a heterogeneous group. As such, population-tailored LRSUGs for specific sub-populations need to be developed to help reduce the particular harms associated with use among these groups. Each of these LRSUGs are important in managing the harms of substance use and addressing these gaps will allow for future LRSUGs to be more effective at addressing the risks of youth substance use.

## Supplementary Information

**Additional file 1: Supplemental Table 1.** Key Search Terms.

## Data Availability

The datasets used and/or analysed during the current study are available from the corresponding author on reasonable request.
