# Supplementary material for: Lower-risk substance use guidelines accessible by youth
Source: Subst Abuse Treat Prev Policy. 2023 Feb 13;18:10. doi: 10.1186/s13011-023-00516-3 (PMC9926657; doi:10.1186/s13011-023-00516-3)
Supplement: Supplementary file 1 — Additional file 1: Supplemental Table 1. Key Search Terms. [file 13011_2023_516_MOESM1_ESM.docx]

**Supplemental Table 1. Key Search Terms**

|  | |
| --- | --- |
| Search Term Used | Drug Name |
| How to Use Cannabis | Cannabis |
| How to Use Cannabis Youth |  |
| Low-Risk Cannabis Use |  |
| Low-Risk Cannabis Use Youth |  |
| Safe Cannabis Use |  |
| Safe Cannabis Use Youth |  |
| Cannabis Guidelines |  |
| Youth Cannabis Guidelines |  |
| How to Use Weed | Weed |
| How to Use Weed Youth |  |
| Low-Risk Weed Use |  |
| Low-Risk Weed Use Youth |  |
| Safe Weed Use |  |
| Safe Weed Use Youth |  |
| Weed Guidelines |  |
| Youth Weed Guidelines |  |
| How to Use Marijuana | Marijuana |
| How to Use Marijuana Youth |  |
| Low-Risk Marijuana Use |  |
| Low-Risk Marijuana Use Youth |  |
| Safe Marijuana Use |  |
| Safe Marijuana Use Youth |  |
| Marijuana Guidelines |  |
| Youth Marijuana Guidelines |  |
| How to Use Alcohol | Alcohol |
| How to Use Alcohol Youth |  |
| Low-Risk Alcohol Use |  |
| Low-Risk Alcohol Use Youth |  |
| Safe Alcohol Use |  |
| Safe Alcohol Use Youth |  |
| Alcohol Guidelines |  |
| Youth Alcohol Guidelines |  |
| How to Use Drinking | Drinking |
| How to Use Drinking Youth |  |
| Low-Risk Drinking |  |
| Low-Risk Drinking Youth |  |
| Safe Drinking |  |
| Safe Drinking Youth |  |
| Drinking Guidelines |  |
| Youth Drinking Guidelines |  |
| How to Use Caffeine | Caffeine |
| How to Caffeine Use Youth |  |
| Low-Risk Caffeine Use |  |
| Low-Risk Caffeine Use Youth |  |
| Safe Caffeine Use |  |
| Safe Caffeine Use Youth |  |
| Caffeine Guidelines |  |
| Youth Caffeine Guidelines |  |
| How to Use Energy Drinks | Energy Drinks |
| How to Use Energy Drinks Youth |  |
| Low-Risk Energy Drink Use |  |
| Low-Risk Energy Drink Use Youth |  |
| Safe Energy Drink Use |  |
| Safe Energy Drink Use Youth |  |
| Energy Drink Guidelines |  |
| Youth Energy Drink Guidelines |  |
| How to Use Prescription Opioids | Prescription Opioids |
| How to Use Prescription Opioids Youth |  |
| Low-Risk Prescription Opioid Use |  |
| Low-Risk Prescription Opioid Use Youth |  |
| Safe Prescription Opioid Use |  |
| Safe Prescription Opioid Use Youth |  |
| Prescription Opioid Guidelines |  |
| Youth Prescription Opioid Guidelines |  |
| How to Use Opioids | Opioids |
| How to Use Opioids Youth |  |
| Low-Risk Opioid Use |  |
| Low-Risk Opioid Use Youth |  |
| Safe Opioid Use |  |
| Safe Opioid Use Youth |  |
| Opioid Guidelines |  |
| Youth Opioid Guidelines |  |
| How to Use Prescription Stimulants | Prescription Stimulants |
| How to Use Prescription Stimulants Youth |  |
| Low-Risk Prescription Stimulant Use |  |
| Low-Risk Prescription Stimulant Use Youth |  |
| Safe Prescription Stimulant Use |  |
| Safe Prescription Stimulant Use Youth |  |
| Prescription Stimulant Guidelines |  |
| Youth Prescription Stimulant Guidelines |  |
| How to Use Stimulants | Stimulants |
| How to Use Stimulants Youth |  |
| Low-Risk Stimulant Use |  |
| Low-Risk Stimulant Use Youth |  |
| Safe Stimulant Use |  |
| Safe Stimulant Use Youth |  |
| Stimulant Guidelines |  |
| Youth Stimulant Guidelines |  |
| How to Smoke | Smoking |
| How to Smoke Youth |  |
| Low-Risk Smoking |  |
| Low-Risk Smoking Youth |  |
| Safe Smoking |  |
| Safe Smoking Youth |  |
| Smoking Guidelines |  |
| Youth Smoking Guidelines |  |
| How to Vape | Vaping |
| How to Vape Youth |  |
| Low-Risk Vaping Use |  |
| Low-Risk Vaping Use Youth |  |
| Safe Vaping |  |
| Safe Vaping Youth |  |
| Vaping Guidelines |  |
| Youth Vaping Guidelines |  |
| How to Use LSD | LSD |
| How to Use LSD Youth |  |
| Low-Risk LSD Use |  |
| Low-Risk LSD Use Youth |  |
| Safe LSD Use |  |
| Safe LSD Use Youth |  |
| LSD Guidelines |  |
| Youth LSD Guidelines |  |
| How to Use Acid | Acid |
| How to Use Acid Youth |  |
| Low-Risk Acid Use |  |
| Low-Risk Acid Use Youth |  |
| Safe Acid Use |  |
| Safe Acid Use Youth |  |
| Acid Guidelines |  |
| Youth Acid Guidelines |  |
| How to Use Magic Mushrooms | Magic Mushrooms |
| How to Use Magic Mushrooms Youth |  |
| Low-Risk Magic Mushroom Use |  |
| Low-Risk Magic Mushroom Use Youth |  |
| Safe Magic Mushroom Use |  |
| Safe Magic Mushroom Use Youth |  |
| Magic Mushroom Guidelines |  |
| Youth Magic Mushroom Guidelines |  |
| How to Use Psilocybin | Psilocybin |
| How to Use Psilocybin Youth |  |
| Low-Risk Psilocybin Use |  |
| Low-Risk Psilocybin Use Youth |  |
| Safe Psilocybin Use |  |
| Safe Psilocybin Use Youth |  |
| Psilocybin Guidelines |  |
| Youth Psilocybin Guidelines |  |

**Additional Files**

File Name: Lower-Risk Substance Use Guidelines for Youth - Guideline Coding Table – (Additional File 1)

File Format: .xlsx

Title of Data: Lower-Risk Substance Use Guidelines for Youth - Guideline Coding Table

Description of data: The dataset which was used to code and generate the information within **Table 2** (LRSUG Digital Assessment Results).
